# External validation of a deep learning-based algorithm for detection of tall cells in papillary thyroid carcinoma: A multicenter study

**DOI:** 10.1016/j.jpi.2024.100366

**Published:** 2024-02-01

**Authors:** Sebastian Stenman, Sylvain Bétrisey, Paula Vainio, Jutta Huvila, Mikael Lundin, Nina Linder, Anja Schmitt, Aurel Perren, Matthias S. Dettmer, Caj Haglund, Johanna Arola, Johan Lundin

**Affiliations:** a Institute for Molecular Medicine Finland – FIMM, University of Helsinki, Tukholmankatu 8, 00290 Helsinki, Finland; b HUSLAB, Department of Pathology, HUS Diagnostic Center, Helsinki University Hospital and University of Helsinki, Haartmaninkatu 3C, 000290 HUS Helsinki, Finland; c Department of Surgery, Helsinki University Hospital, Haartmaninkatu 4, 00290 Helsinki, Finland; d Institute of Tissue Medicine and Pathology, University of Bern, Murtenstrasse 31, 3008 Bern, Switzerland; e Department of Pathology, University of Turku, Turku University Hospital, Kiinamyllykatu 10, 20520 Turku, Finland; f The Global Health & Migration Department of Women’s and Children’s Health, Uppsala University, 75185 Uppsala, Sweden; g Institute of Pathology, Klinikum Stuttgart, Kriegsbergstraße 60, 70174 Stuttgart, Germany; h Research Programs Unit, Translational Cancer Medicine, University of Helsinki, Haartmaninkatu 4, 00290 Helsinki, Finland; i Department of Global Public Health, Karolinska Institutet, Norrbackagatan 4, 17176 Stockholm, Sweden; j iCAN Digital Precision Cancer Medicine Flagship, Helsinki, Finland

**Keywords:** Papillary thyroid carcinoma, Artificial intelligence, Digital pathology, Deep learning

## Abstract

The tall cell subtype (TC-PTC) is an aggressive subtype of papillary thyroid carcinoma (PTC). The TC-PTC is defined as a PTC comprising at least 30% epithelial cells that are three times as tall as they are wide. In practice, this definition is difficult to adhere to, resulting in high inter-observer variability. In this multicenter study, we validated a previously trained deep learning (DL)-based algorithm for detection of tall cells on 160 externally collected hematoxylin and eosin (HE)-stained PTC whole-slide images. In a test set of 360 manual annotations of regions of interest from 18 separate tissue sections in the external dataset, the DL-based algorithm detected TCs with a sensitivity of 90.6% and a specificity of 88.5%. The DL algorithm detected non-TC areas with a sensitivity of 81.6% and a specificity of 92.9%. In the validation datasets, 20% and 30% TC thresholds correlated with a significantly shorter relapse-free survival. In conclusion, the DL algorithm detected TCs in unseen, external scanned HE tissue slides with high sensitivity and specificity without any retraining.

## Introduction

The tall cell subtype of papillary thyroid carcinoma (TC-PTC) is an aggressive subtype compared to classical PTC requiring more aggressive treatment.^1^^,^^2^ The World Health Organization’s (WHO) Classification of Tumors defines the TC-PTC as a tumor containing at least 30% epithelial cells that are three times as tall as they are wide often with abundant eosinophilic cytoplasm.^3^ However, the task of identifying and quantifying TCs within PTCs is laborious and prone to subjectivity which results in significant inter-observer variability.^4^^,^^5^ Indeed, the TC percentage required for a tumor to be regarded as a TC-PTC varies in the literature from 10% tall cells^6^ to over 50%.^7^ Others have reported on PTC with TC like features, i.e., tumors containing some TCs but not enough to meet the TC-PTC threshold. These tumors have been shown to be more aggressive and correlate with a poor prognosis.^8^^,^^9^

Deep learning (DL) algorithms have proven promising for a wide range of applications in tissue sample analysis.^10^ In thyroid cancer, DL algorithms have previously been used for tasks such as analysis of inter-operative frozen section samples of thyroid nodules,^11^ gene expression identification in neoplasms with papillary-like nuclear features,^12^ and segmentation of tumor infiltrating lymphocytes.^13^ Previously, a DL-based algorithm has been trained and tested for TC area detection and quantification showing a correlation between reduction in relapse-free survival (RFS) for patients with a TC percentage above 30%.^14^

Despite the proven success of DL in various image analysis tasks, only a few DL algorithms have been clinically deployed so far. An important reason for this is the challenge of ensuring that the performance of the tested algorithm transfers to new, unseen datasets, i.e., algorithm generalizability.^15^^,^^16^ The trained DL models are often validated on internal data which might not capture the variability of sample processing, staining, and digitization occurring between laboratories. Therefore, validating the trained algorithm on external datasets is crucial for performance evaluation but is still often an overlooked step in the process.^17^^,^^18^

In the present study, we validate the performance of a previously trained DL algorithm^14^ for TC scoring. Our aim was to evaluate the generalizability and robustness of the DL algorithm with regards to identification of TCs when applied to external whole-slide image (WSI) dataset; one originating from University of Bern, Switzerland, the other from Auria Biobank, Turku, Finland. Also, we evaluated the association between the TC score and survival.

## Materials and methods

### Training of the deep learning-based algorithm

The DL algorithm to be assessed in the current study was trained on a dataset comprising of 100 WSIs from 100 individual patients; 70 from a previously studied dataset from Helsinki University Hospital and 30 WSIs downloaded from The Cancer Genome Atlas.^19^ The trained DL algorithm consisted of two algorithms run in sequence; first, an algorithm segments the tumor tissue which is fed as input to the second algorithm quantifying the TC and non-TC areas. The TC algorithm was trained on a total of 2674 manual annotations of regions of interest within the 100 WSIs in the training dataset as previously described.^14^

### External whole-slide image datasets

#### The Auria Biobank dataset

The first external dataset used in the study was obtained via the Auria Biobank which stores samples and data from patients treated in the Turku University Hospital region. A total of 81 patients treated for PTC between 2003 and 2013 were obtained and 18 of these patients experienced an adverse outcome. An adverse outcome was defined as at least two local recurrences (histologically confirmed or elevation in serum thyroglobulin levels during follow-up), distant metastasis, or death from PTC. All material was re-evaluated by two experienced endocrine pathologists (PV, JH) and one formalin-fixed and paraffin-embedded (FFPE) tissue block containing the most representative tumor regions was selected for each patient. New sections were freshly cut and fixed on glass slides and stained with hematoxylin and eosin (HE) according to standard procedure. The HE stained samples were then digitized using a WSI scanner (Pannoramic 250 FLASH 3DHISTECH Ltd., Budapest, Hungary) equipped with a plan-apochromat at objective 20× (NA 0.8), a CMOS camera (Adimec Q-12A-180, Eindhoven, The Netherlands) with a 1.6 adapter which gives a pixel size of 0.24 μm*.* The WSIs were then imported to an image management platform (Aiforia Hub, Aiforia Technologies Oy, Helsinki, Finland). Eight WSIs were dropped due to poor staining quality or lack of tumor material. The final dataset consisted of 73 WSIs; 17 patients with an adverse outcome and 56 control cases (Table 1).

**Table 1:** Patient characteristics of the Auria Biobank dataset.

Characteristics	Adverse outcome (*n*=17)	Control group (*n*=56)	*p*-value
Female	9 (47%)	11 (20%)	0.06
Male	8 (53%)	45 (80%)	
Mean age at diagnosis	54.5 (SD 15.2)	50.0 (SD 17.8)	0.08
Nodal metastases	14 (82%)	13 (23%)	<0.001
Primary distant metastases	1 (6%)	0 (0%)	0.26
Relapse	17 (100%)	17 (30%)	<0.001
Stage of tumor - T1 - T2 - T3 - T4 Unclear	3 (18%) 4 (23%) 7 (41%) 2 (12%) 1 (6%)	21 (38%) 18 (32%) 11 (20%) 1 (2%) 5 (8%)	0.06
Primary RAI	17 (100 %)	53 (95%)	1.00
Median algorithm TC score	32.5% (SD 12.6)	25.4% (SD 14.8)	0.10
Died of PTC	3 (17%)	0 (0%)	0.01

#### The Bern dataset

**Fig. 1:**
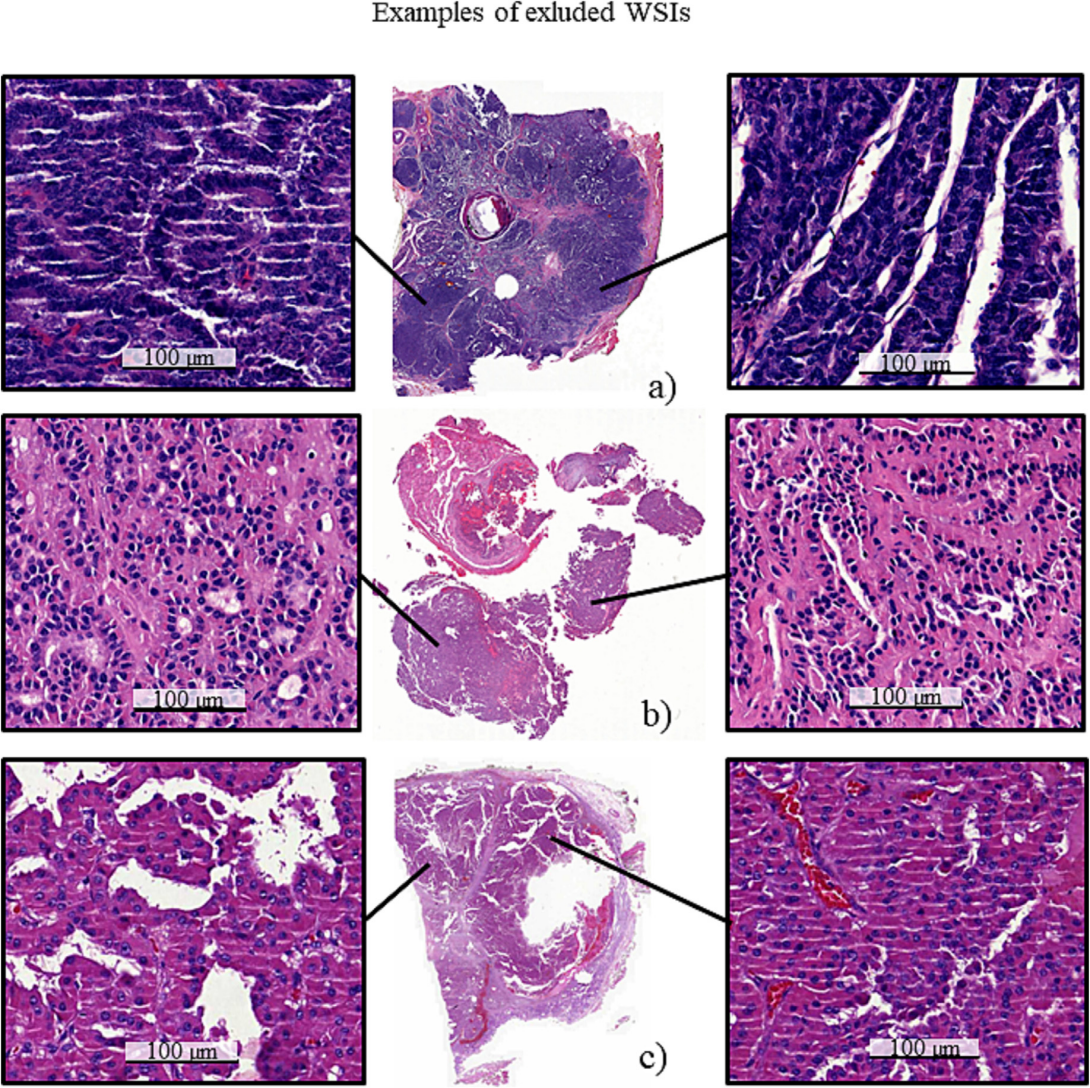
Examples of excluded whole-slide images (WSIs). WSIs were excluded because of e.g., too thick section resulting in very dark samples (a), tissue sample damage resulting in poor quality of the morphology (b, c).

The second external datasets used in the study was a previously described PTC series^6^^,^^20^ originally consisting of 125 patients. All patients had undergone surgery for primary thyroid cancer between 1990 and 2006. Tissue samples from 100 patients from the original cohort was acquired and all FFPE tissue blocks from each patient were revisited. The remaining 25 patients were excluded from this study due to lack of tissue samples. One representative tissue block was selected for each of the 100 included patients. New tissue sections were cut of the representative tissue blocks, stained with HE and digitized using a WSI scanner (Pannoramic 250 FLASH 3DHISTECH Ltd., Budapest, Hungary) equipped with a plan-apochromat at objective 20×, Camera type CIS VCC-FC60FR19CL with a pixel size of 0.24 μm/pixels and a 1.6 adapter)*.* The digitized WSIs were then uploaded to an image management platform (Aiforia Hub, Aiforia Technologies Oy, Helsinki, Finland). At this phase, 13 additional WSIs were excluded due to poor staining- or scanning quality, or lack of representative areas of tumor tissue on the digital slide (Fig. 1). Thus, the final dataset consisted of 87 WSIs (Table 2).

**Table 2:** Patient characteristics of the Bern dataset.

Characteristics	PTC patients (*n*=87)
Female^a^	61 (70%)
Male^a^	21 (24%)
Mean age at diagnosis^a^ (*n*=82)	51.5 (SD 19.7)
Nodal metastases	14 (16%)
Primary distant metastases	4 (5%)
Relapse	8 (9%)
Stage of tumor - T1 - T2 - T3 - T4 Unclear	25 (28%) 19 (22%) 24 (28%) 9 (10% 10 (12%)
Median algorithm TC score	11.6% (SD 9.3)
Died of PTC	0

a Full data unavailable.

### Algorithm performance evaluation

**Fig. 2:**
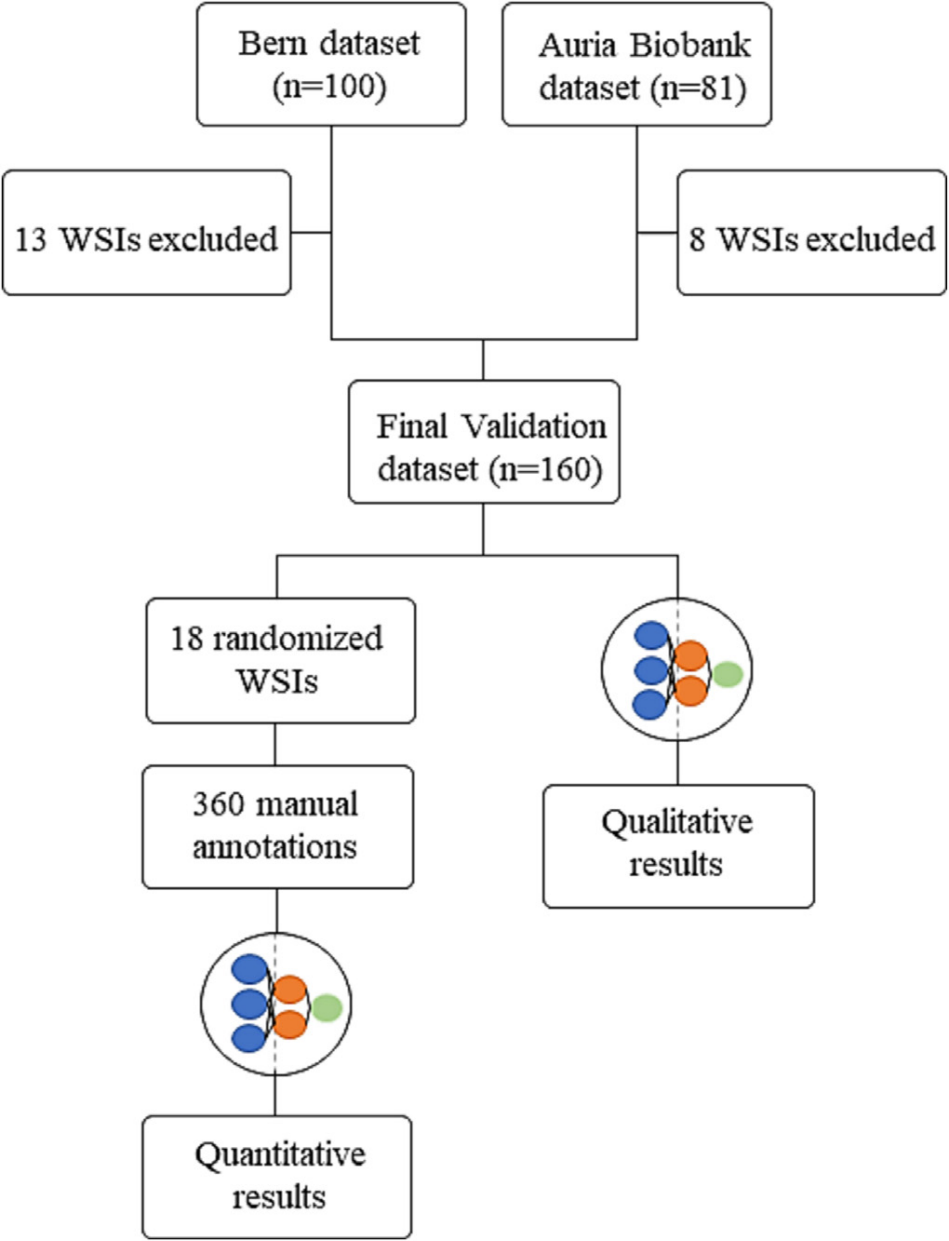
Consort flowchart of algorithm validation. The performance of the trained deep learning-based algorithm for tall cell scoring was evaluated both quantitatively and qualitatively. For quantitative performance analysis, nine whole-slide images (WSIs) per external dataset were randomly selected. Twenty manual annotations of regions of interest were created per randomized WSI on which the tall cell algorithm was evaluated. All included WSIs were analyzed by the trained deep learning algorithm and visually evaluated as a qualitative performance evaluation.

For quantitative assessment of the trained DL algorithm, we randomly selected 9 WSIs per external dataset resulting in a total of 18 WSIs (Fig. 2). One researcher (SS) manually annotated 20 regions of interest per randomized WSI blinded to the algorithm output. This resulted in a total of 360 manually annotated regions of interest on which the TC algorithm was quantitatively evaluated (Fig. 2). The total area of the annotated regions of interest was 4.16 mm^2^ which averaged to an area of 0.016 mm^2^ per manual annotation. Furthermore, all WSIs included in the study were analyzed with the DL algorithm and the heatmaps indicating TC and non-TC areas were evaluated qualitatively by the researchers.

### Statistical analysis

Statistical analysis was performed using a statistical software package (Stata 17.0 for Mac Stata Corp., College Station, TX). The number of manual annotations needed was calculated assuming a sensitivity of 90%, TC prevalence of 10%, width of confidence interval of 10%, and a confidence level of 95% resulting in a minimum of 346 manual annotations. The performance metrics reported for the DL-based algorithm was sensitivity (recall), precision (positive-predictive value, PPV), and F1 score (the harmonic means of precision and recall). The statistical distribution of the samples according to their TC score were analyzed using the Mann–Whitney *U* test. The statistical analysis employed Fisher’s exact test to evaluate group differences for nominal variables. RFS was defined as the time between the primary operation until relapse or end of follow-up. We employed the Kaplan–Meier method to estimate survival probabilities and generate survival curves. Differences in survival between groups were assessed using the logrank test. A *p*-value of lower than 0.05 was considered as statistically significant and two tailed tests were used.

## Results

### Algorithm performance

**Fig. 3:**
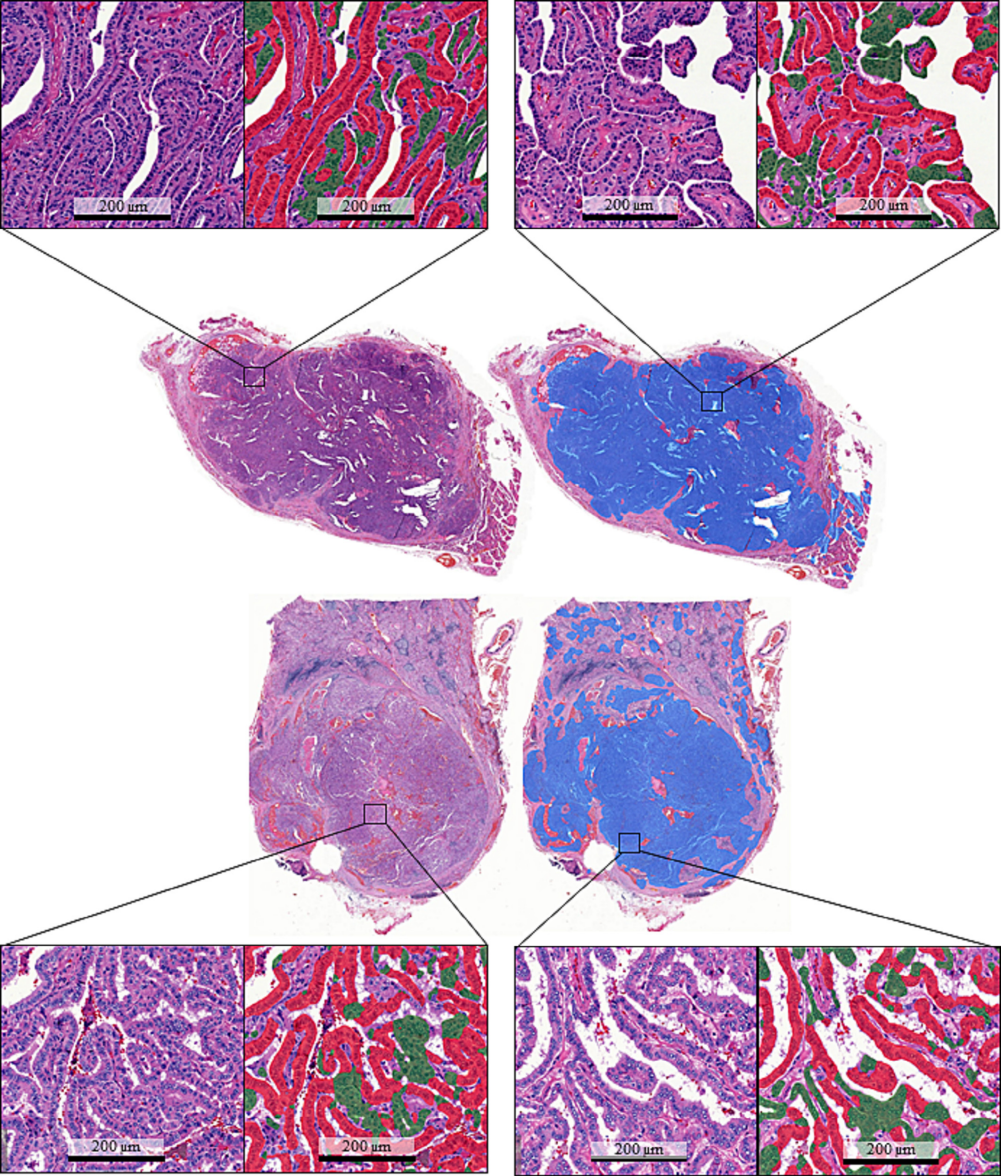
Algorithm structure and results. Two good quality examples from the external validation datasets. The trained deep learning-based algorithm consisted of two algorithms. First, one algorithm segmented tumor tissue (blue). A sequential algorithm then segments tall cell epithelium (red) from non-tall cell epithelium (green) and a tall cell score was then calculated.

**Fig. 4:**
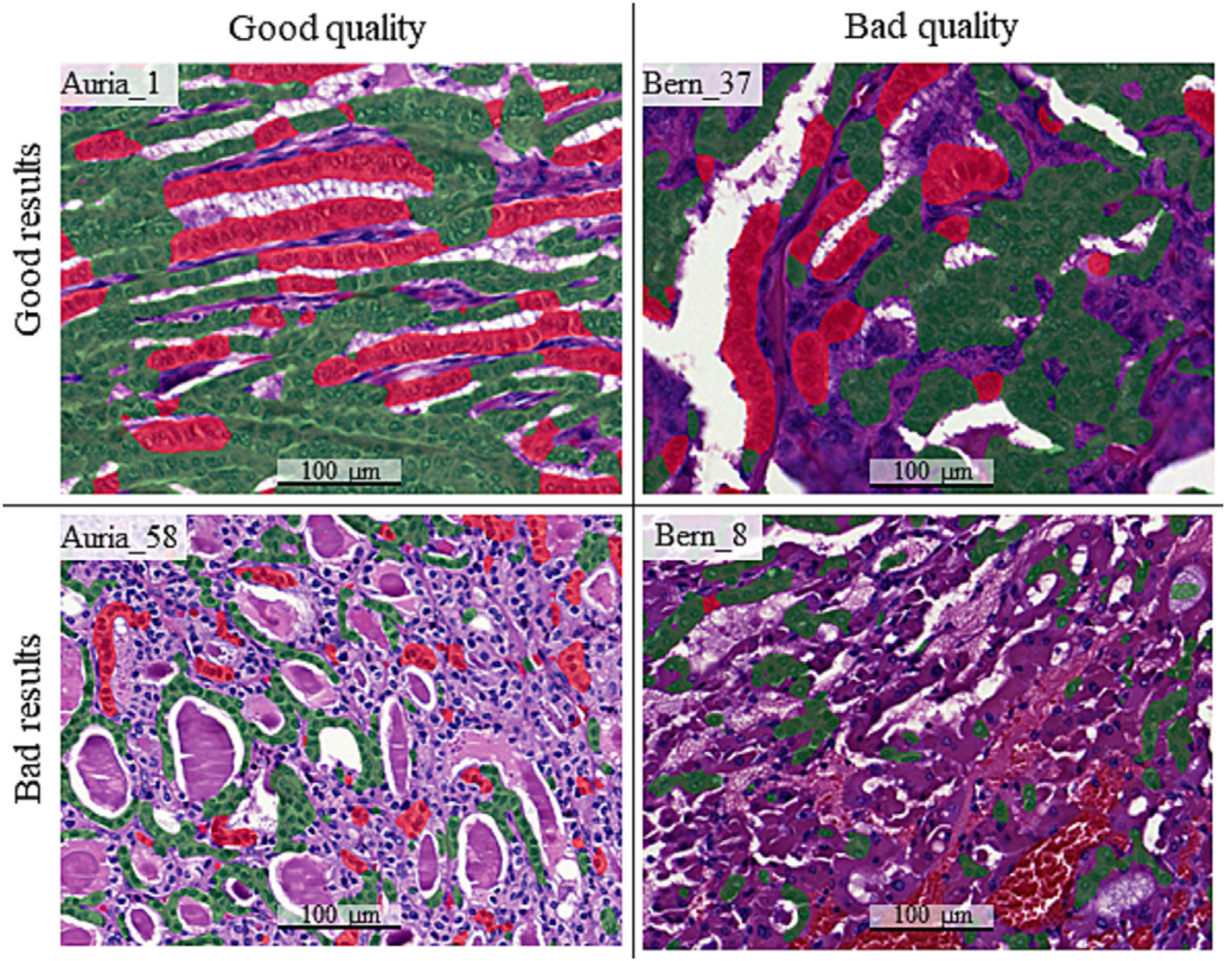
Example images. The trained tall cell (TC) deep learning-based algorithm was validated on two external papillary thyroid carcinoma datasets. The whole-slide images (WSIs) were of varying quality; some thick sections that result in a dark sample and some tissue sections with scanning artefacts or damaged in the staining process. Overall, the algorithm performed well on high-quality sections, and worse on sections of lower quality. In a few WSIs, the algorithm performed poorly despite a rather good tissue slide quality. TC regions registered by the algorithm is highlighted with red and registered non-TC areas is highlighted with green.

In the 360 manual annotations for quantitative performance evaluation, the DL algorithm segmented TC regions with a sensitivity of 91% (95% CI [86–95%]), a positive-predictive value (precision) of 89% (95% CI [83–94%]), and an F1 score of 88% (95% CI [85–94%]). Non-TC areas were segmented with a sensitivity of 82% (95% CI [77–86%]), a PPV of 93% (95% CI [89–97%]), and an F1 score of 87% (95% CI [82–92%]) (Fig. 3). The WSIs included in the study and the algorithm results (Fig. 4) can be viewed via the following URL: *https://tinyurl.com/TC-Algorithm*.

### TC score and survival

In the Auria Biobank validation dataset, the median TC score for the adverse outcome group was 32.5% (range 11.5–56.7%, SD 12.6) and for the control group 25.4% (range 1.13–55.5%, SD 14.8) but the difference was not significant (*p*=0.10). No statistically significant distribution between adverse vs control outcome groups was observed when studying TC thresholds of 10%, 20%, 30%, 40%, and 50% (*p*=0.19, *p*=0.09, *p*=0.10, *p*=1.00, and *p*=0.66, respectively).

In the Bern validation dataset, the median TC score was 11.6% (SD=9.3, range 0.59–46.6%).

**Fig. 5:**
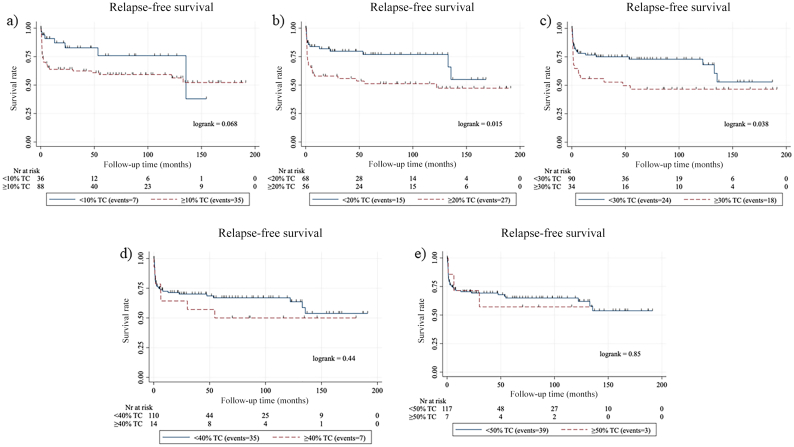
Survival analysis. Kaplan–Meier curves for relapse-free survival among patients with papillary thyroid carcinoma according to five tall cell percentage thresholds: (a) 10%, (b) 20%, (c) 30%, (d) 40%, and (e) 50% using a deep learning-based algorithm.

**Supplementary Figure S1:**
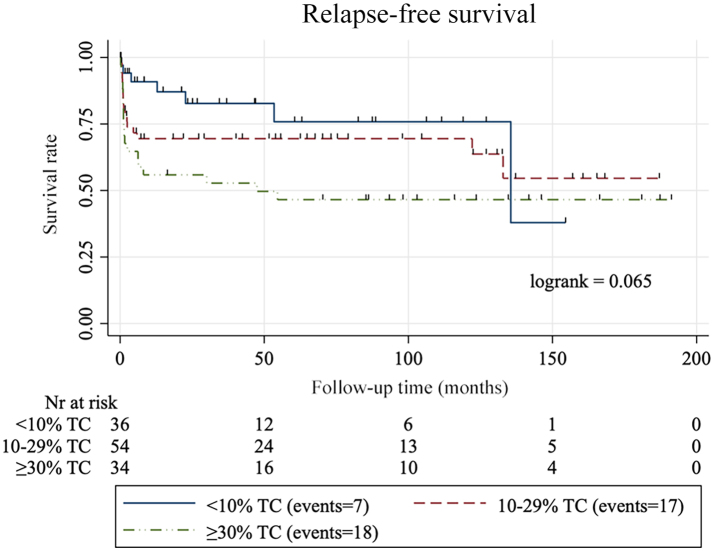
Kaplan-Meier curves for relapse-free survival among patients with papillary thyroid carcinoma according to tall cell (TC) percentage thresholds: <10% TC, 10-29% TC, and ≥30% TC.

For log-rank survival analysis, the two validation datasets were combined. Five TC thresholds were studied, 10%, 20%, 30%, 40%, and 50%. We found a significant association between a higher TC score and a reduced RFS using the thresholds 20% and 30% (*p*=0.015 and *p*=0.038, respectively), but not for 10%, 40%, or 50% TC thresholds (*p*=0.068, *p*=0.44, and *p*=0.85, respectively) (Fig. 5). When grouping the samples based on their TC score into three groups of <10% TC, 10–29% TC, and ≥30% TC, we found no significant decrease in RFS between the groups (Supplementary figure 1).

## Discussion

The TC-PTCis more aggressive than the classical subtype and should be treated accordingly. In this multicenter study, we validate a previously trained DL-based algorithm^14^ for tall cell quantification on two externally collected and prepared datasets. The DL-based algorithm managed to segment areas containing TCs in PTC WSIs with high specificity and sensitivity without any retraining or support training (*https://tinyurl.com/TC-Algorithm*). Survival analysis demonstrated a correlation between a reduction in RFS for TC thresholds of 20% and 30%.

In quantitative performance evaluation on 360 manual annotations in 18 WSIs from the external datasets, the DL algorithm had a sensitivity of 85% and a PPV of 89% for TC regions and 82% sensitivity and 93% PPV for non-TC regions. This is a relatively small drop in performance compared to the original study demonstrating a 94% sensitivity and 95% PPV for TC regions and 91% sensitivity and 94% PPV for non-TC regions in internal validation.^14^ This shows that the performance is good on new, unseen datasets without any retraining of the model. When visually evaluating the results, we concluded that the TC algorithm performed well on WSIs of high quality, whereas the performance suffered on thick and dark slides or slides containing staining and/or scanning artifacts. This is expected since we used supervised learning in the training of the TC algorithm where the input data were manually drawn annotations on high-quality regions. Because it is important to label the training data carefully and as accurately as possible, we did not include areas in which the annotator was not able to clearly distinguish TCs from non-TC regions. The results are presented and publicly available for further visual assessment on a digital platform (*https://tinyurl.com/TC-Algorithm*).

Because PTC has an overall very good prognosis with only few deaths from disease, we defined an adverse outcome as at least two relapses, primary distant metastases or during follow-up, or death from PTC. Despite this, for the Auria Biobank dataset, we only managed to include 17 adverse outcome cases in the final validation dataset. It is important to note that this broadened definition might have allowed more indolent cases of PTC to be included in the adverse outcome group. The adverse outcome group did have a higher median TC score of 32.5% compared to 25.4% in the control group. The difference was not statistically significant (*p*=0.10) which at least partly may be explained by the low number of cases.

For survival analysis, we combined the two external datasets to increase the number of cases. Two analyzed thresholds, a TC score over 20% TC and 30% TC thresholds correlated with a reduction in RFS (*p*=0.015 and *p*=0.038, respectively) which is in line with the WHO suggestion of a 30% TC cut-off for TC-PTC.^3^ Also, these findings are in line with the notion that all cases with more than 10% but less than 30% TCs, i.e., PTC with tall cell features, have a worse prognosis than the conventional subtype as has been reported previously.^8^^,^^9^^,^^21^

An absence of proper validation of trained algorithms is a common problem and many studies reporting well performing models are of high risk of bias.^22^^,^^23^ The lack of rigorous evaluation using external data is particularly lacking. One meta-analysis showed that only 31 studies out of 516 eligible published studies performed external validation.^24^ The proposed DL model in this study performed with a high sensitivity and specificity in external validation. We focused on improving the generalizability of our model in the training phase already by using a multicenter training dataset; 70 WSIs from a dataset from Helsinki, Finland and 30 from the TCGA database.^14^ Furthermore, in the training process, we utilized morphological augmentations such as rotation variation of scale, shear distortion, and aspect ratio. We also deployed stain color augmentations by altering contrast, white balance, and luminance to improve the generalizability of the trained model.

A strength of the current study is the external datasets originating from two different centers. This allows us to test the generalizability of the trained TC algorithm as it encounters variations in staining and scanning compared to the training dataset. However, the size of the datasets could be considered a limitation and limits the possibility to perform extensive outcome analyses.

To our knowledge, the proposed and tested method for TC segmentation is the first of its kind and the novelty of the proposed method could be considered a strength. However, it is worth noting that other features and prognostic factors than TC percentage should be considered by the pathologist when making an evaluation. Important well-known prognostic factors include a higher age at diagnosis,^25^ extrathyroidal extensions,^26^^,^^27^ and tumor size.^26^ The prognosis is also determined by the clinical extent of the disease. Indeed, the 10-year survival rate for stage I disease is over 99%, whereas the survival rate for stage IV disease is under 50%.^28^ Morphological features and histological subtypes must also be considered, and one should also be aware of other subtypes of PTC with an adverse outcome e.g., the columnar cell subtype.^3^

In one study, the outcomes of groups of patients were compared to varying TC levels and concluded that an aggressive disease is largely driven by classical clinicopathological features and that clinical management should not be based on tall cell percentage alone.^29^ However, the TC scoring for this study was assumably done by visual evaluation using traditional microscopy which is known to be affected by subjectivity with large inter-observer variability.^4^ The proposed DL-based algorithm is a tool that should be used in assisting pathologists in TC scoring. Other factors than the TC score need to be considered in the evaluation and the tool should therefore not be used for replacing pathologists but to enhance and provide a more objective way to determine a TC score for a tumor.

We conclude that our DL-based algorithm generalizes well in TC scoring when applied to externally collected datasets and segments TC regions with a high sensitivity and specificity. In future studies, this method for TC scoring should be evaluated on a prospective PTC cohort and should be evaluated to see how it could assist pathologists in diagnosing TC-PTC.

The following are the supplementary data related to this article.

## Author Contributions

Conceptualization, S.S., J.A., C.H., J.L.; Methodology, S.S., J.A., C.H., J.L.; Formal analysis, S.S., J.H., P.V., S.B., A.S.; Data Curation, M.L., J.H., P.V., S.B., A.S.; Writing – Original Draft Preparation, S.S.; Writing – Review & Editing J.A., C.H., J.L., M.D., A.P.; Visualization, S.S., M.L.; Supervision, J.A., C.H., J.L.; Project Administration J.A., C.H., J.L.; Funding Acquisition, S.S., N.L., J.A., C.H., J.L.

## Funding

The study was supported by the Sigrid Juselius Foundation, 10.13039/100010135Finska Läkaresällskapet, 10.13039/100010116Medicinska Understödsföreningen Liv och Hälsa, 10.13039/100010119Stiftelsen Dorothea Olivia, Karl Walter och Jarl Walter Perkléns minne, iCAN Digital Precision Cancer Medicine Flagship, and HiLIFE Helsinki Institute of Life Sciences.

## Institutional Review Board Statement

Auria Biobank collects samples from patients in the Turku University Hospital district in Finland. The biobank operates in accordance with the Finnish Biobank Act (688/2012) and is licensed by the National Supervisory Authority of Welfare and Health (Valvira). This study was approved by the Scientific Steering Committee of Auria Biobank. The study was conducted in accordance with the Declaration of Helsinki.

The Bern dataset has previously been reported and usage was approved by the cantonal Ethics committee (KEK BE 2018 01657). The patient material was de-identified prior to electronic transferal.

## Informed Consent Statement

The Auria Biobank dataset originated from Auria Biobank and the samples were collected in accordance with the Finnish Biobank Act (688/2012). The samples are collected by the biobank via informed consent. The Bern dataset was used according to the Ethics-permission (KEK BE 2018 01657)

## Declaration of competing interest

Johan Lundin is a co-founder and board member, and Mikael Lundin is a co-founder and director of concept design at Aiforia Technologies Oy, Helsinki Finland.

## Data Availability

All whole-slide images included in the study can be studied by the reader in further detail via the following URL: *https://tinyurl.com/TC-Algorithm*.
